# Oral fosfomycin for treatment of acute bacterial prostatitis caused by multidrug-resistant Enterobacterales

**DOI:** 10.1128/spectrum.02136-23

**Published:** 2023-09-22

**Authors:** Joaquin Burgos, Yannick Hoyos-Mallecot, Carles Ferre-Losa, Maider Arando, Arnau Monforte, Tomas Pumarola, Ibai Los-Arcos, Vicenç Falcó

**Affiliations:** 1 Infectious Diseases Department, Hospital Universitari Vall d'Hebron. Vall d'Hebron Research Institute (VHIR). Universitat Autònoma de Barcelona, Barcelona, Spain; 2 Microbiological Department, Hospital Universitari Vall d'Hebron. Vall d'Hebron Research Institute (VHIR). Universitat Autònoma de Barcelona, Barcelona, Spain; 3 Emergency Department, Hospital Universitari Vall d'Hebron. Vall d'Hebron Research Institute (VHIR). Universitat Autònoma de Barcelona, Barcelona, Spain; Johns Hopkins Hospital, Baltimore, Maryland, USA

**Keywords:** fosfomycin-tromethamine, acute prostatitis, multidrug resistance

## Abstract

To assess the feasibility of oral fosfomycin-tromethamine (FT) for the management of acute bacterial prostatitis (ABP) caused by multidrug-resistant (MDR) Enterobacterales. An observational study of adult patients diagnosed with ABP from Vall d’Hebron University Hospital (Barcelona, Spain), treated with oral FT. The primary outcome was clinical cure defined as symptom relief at the control visit, 2–4 weeks post-end of treatment. Secondary outcomes included microbiological cure, relapse, and adverse events related to the treatment. Eighteen patients with ABP caused by Enterobacterales (15 *Escherichia coli* and three *Klebsiella pneumoniae*) were included. Microorganisms were MDR bacteria [14 extended-spectrum beta-lactamase (ESBL) producers and two carbapenemase producing *K. pneumoniae*]. Patients received treatment with FT 3 g/48 hours during a median of 14 days (Q25–Q75, 12–17.75). Fifteen patients received a lead-in phase of intravenous suitable antimicrobial during a median of 7 days (Q25–Q75, 3.75–8). No patient had to stop treatment due to adverse events, and the only side effect reported in two patients was diarrhea. Clinical cure was achieved in all (18/18) patients and microbiological cure in 11/12 patients. After a median of follow-up of 5 months (Q25–Q75, 2–11), 2/18 patients relapsed with an orchitis and a new episode of ABP. FT is an attractive step-down therapy for ABP in patients with resistance or side effects to first-line drugs. The availability of oral treatment could reduce the use of the carbapenems, with a benefit in the quality of life of the patient, health costs, and an ecological impact.

**IMPORTANCE:**

We present a brief but largest and interesting experience in which we use fosfomycin-tromethamine (FT) for the treatment of acute bacterial prostatitis (ABP) due to multiresistant bacteria. Our study provides new data that help to consider FT as a plausible alternative for treating ABP in patients with resistance or side effects to first-line drugs. The availability of an alternative oral treatment to avoid the use of the carbapenems could have important benefits in terms of quality of life of the patient, health costs, and an ecological impact.

## INTRODUCTION

Acute bacterial prostatitis (ABP) is a common illness and a frequent cause of primary care consultation and hospital admission (1). This illness is also considered a cumbersome-to-treat infection owing to limited antibiotic choices and poor drug distribution in prostatic tissue (2). Fluoroquinolones are the antimicrobials recommended as the first line for the management of ABP. Unfortunately, there is an increased prevalence of challenging antibiotic resistant microorganisms. Nowadays, the rate of resistance to quinolones exceeds 30%, and up to 10% of urinary tract infections (UTIs) are caused by extended-spectrum beta-lactamase (ESBL) producing Enterobacterales (3). In this situation, the therapeutic options are limited and often concern intravenous treatments, frequently with carbapenems.

Oral fosfomycin-tromethamine (FT) could be an alternative to carbapenems in this type of infection. Recent clinical data in women support its efficacy in febrile UTIs (4). In the prostate, pharmacokinetic data show an acceptable bioavailability and adequate intra-prostatic diffusion of FT acquiring therapeutic rate (5). Furthermore, FT remains active in 90% of microorganisms causing ABP, including ESBL (6).

In our facility, we have used oral FT as step-down treatment in selected patients with ABP caused by multidrug-resistant (MDR) microorganisms. The aim of this study is to assess the feasibility of oral FT for the management of ABP caused by MDR Enterobacterales.

## MATERIALS AND METHODS

### Study population and setting

We conducted an observational prospective study of adult patients (aged >16 years) diagnosed with ABP from January 2021 to April 2023 at Vall d’Hebron University Hospital (Barcelona, Spain) and who started oral FT for the management of the illness (PR(AG)626/2020).

Demographic data, clinical presentation, microbiological data, antimicrobial therapy, and outcomes were recorded from each episode. Patient’s follow-up was carried out according to the usual clinical practices.

### Outcome measures and definitions

ABP was defined when the following criteria were fulfilled: (i) abrupt presentation of voiding symptoms (irritative and/or obstructive), (ii) temperature >37.8°C, (iii) presence of bacteriuria in a clean-catch mid-stream urine specimen, and (iv) absence of data suggestive of pyelonephritis (costovertebral angle tenderness).

We excluded cases of fever presenting within 24  hours of urinary tract manipulation and cases suggestive of chronic bacterial prostatitis (CBP).

The primary outcome assessed was clinical cure defined as being alive with symptom relief at the control visit, 2–4 weeks post-end-of-treatment. Secondary outcomes included microbiological cure, defined as sterile control urine taken at 2–4 weeks after treatment; relapse, defined as recurrence with the same microorganism and antibiotic susceptibility pattern during the next 6 months of follow-up; and adverse events related to the treatment.

### Culture, identification, and antibiotic susceptibility

Urine samples were collected in a vacutainer and cultured in BBL CHROMagar Orientation (CO; Becton Dickinson, Cockeysville, Md.). Cultures were incubated for 24–48 hours at 35–37°C. Suggestive colonies of uropathogens were identified by mass spectrometry (MALDI-TOF, Vitek MS system, bioMérieux, Spain). Antibiotic susceptibility testing was performed by the automated Vitek2 system (BioMerieux, France). Minimum inhibitory concentrations (MICs) were interpreted according to criteria established by EUCAST 2023 guidelines (7).

## RESULTS

### Baseline characteristics

Over the study period**,** 18 patients were included. The median age of patients was 68.9 years (Q25–Q75, 57.6–78.9); four (22.2%) had diabetes mellitus and 14 (87.8%) had urologic pathology.

### Etiology of acute prostatitis and antibiotic susceptibility

The isolated pathogens included *Escherichia coli* (*n* = 15) and *Klebsiella pneumoniae* (*n* = 3). All isolates were MDR bacteria and resistant to quinolones and cotrimoxazole, and also all have some grade of resistance to beta-lactams: 14 isolates were ESBL producers, 2 cases a carbapenemase producing *K. pneumoniae* (1 KPC and 1 OXA-48 type), and in the remaining 2 cases, one was resistant to amoxicillin-clavunate and the other resistant both to amoxicillin-clavunate and cefuroxime (Table 1).

**TABLE 1:** Baseline data, treatment, and outcomes of ABP-treated patients with fosfomycin-tromethamine^*c*^

Baseline				Treatment			Success		
Patient	Age	Urologic pathology	Microorganism and resistance	Fosfomycin MIC^*a*^	Active Ev suitable antimicrobial	Oral FT	Clinical cure	Microbiological cure	Relapse
1	77	BPH^*b*^	*K. pneumoniae*, OXA-48 like	32 mg/L	Ceftaz-avi, 3 days	FT, 12 days	Yes	Yes	No
2	49	Urinary incontinence	*E. coli*, ESBL	≤16 mg/L	Ertapenem, 4 days	FT, 12 days	Yes	No	Yes, at 4 weeks
3	72	BPH	*E. coli*, ESBL	≤16 mg/L	Ertapenem, 8 days	FT, 12 days	Yes	Yes	No
4	61	BPH	*E. coli*, ESBL	≤16 mg/L	Ertapenem, 10 days	FT, 11 days	Yes	NA	No
5	57	None	*E. coli*, ESBL	≤16 mg/L	Ertapenem, 7 days	FT, 14 days	Yes	Yes	No
6	57	BPH	*K. pneumoniae*, ESBL	32 mg/L	Ertapenem, 7 days	FT, 14 days	Yes	Yes	No
7	83	BPH	*E. coli*, ESBL	≤16 mg/L	Ertapenem, 9 days	FT, 10 days	Yes	Yes	No
8	59	BPH	*K. pneumoniae*, KPC	≤16 mg/L	None	FT, 20 days	Yes	Yes	No
9	81	BPH	*E. coli*, ESBL	≤16 mg/L	None	FT, 21 days	Yes	NA	Yes, at 5 weeks
10	69	BPH	*E. coli,* resistant A/C	≤16 mg/L	Ceftriaxone, 5 days	FT, 35 days	Yes	Yes	No
11	75	BPH	*E. coli*, resistant A/C and cefuroxime	≤16 mg/L	None	FT, 41 days	Yes	NA	No
12	77	Urethral stricture	*E. coli*, ESBL	≤16 mg/L	Ertapenem, 7 days	FT, 14 days	Yes	Yes	No
13	89	BPH	*E. coli*, ESBL	≤16 mg/L	Ertapenem, 7 days	FT, 14 days	Yes	NA	No
14	33	None	*E. coli*, ESBL	≤16 mg/L	Ertapenem, 4 days	FT, 14 days	Yes	NA	No
15	68	BPH	*E. coli*, ESBL	≤16 mg/L	Ertapenem, 5 days	FT, 17 days	Yes	Yes	No
16	93	Urinary incontinence	*E. coli*, ESBL	≤16 mg/L	Ertapenem, 8 days	FT, 13 days	Yes	NA	No
17	36	None	*E.coli*, ESBL	≤16 mg/L	Ertapenem, 7 days	FT, 14 days	Yes	Yes	No
18	67	None	*E.coli*, ESBL	≤16 mg/L	Ertapenem, 10 days	FT, 14 days	Yes	Yes	No

^*a*^ MIC testing was performed by the automated Vitek2 system (BioMerieux, France).

^*b*^ BPH, benign prostatic hyperplasia; ESBL, extended-spectrum beta-lactamase; Ev, intravenous; FT, fosfomycin-trometamol; Cefta-avi, ceftazidime-avibactam; A/C, amoxicillin-clavunate; NA, not available; MIC, minimum inhibitory concentrations.

^*c*^ Dose: 1g/24 hours of ertapenem, 2g/8 hours of cefta-avi and 2g/24 hours de ceftriaxone.

### Antimicrobial therapy

Only six patients had an active empirical treatment. According to the susceptibility pattern of isolate, 15 patients received an intravenous suitable antimicrobial (13 ertapenem, 1 ceftazidime-avibactam, and 1 patient ceftriaxone) during a median of 7 days (Q25–Q75, 3.75–8). After that, step-down therapy with FT was undertaken. In three patients, they directly administered FT without previous active treatment. All patients included received FT 3 g every 48 hours during a median of 14 days (Q25–Q75, 12–17.75). The median of days of overall suitable antimicrobial was 21 (Q25–Q75, 19.75–22.5). They attributed no serious side effects to FT, and no patient had to stop treatment due to adverse events. Diarrhea was the only side effect reported and occurred in two patients.

Regarding efficacy, all patients presented a resolution of urinary symptoms during treatment. All patients achieved clinical cure, assessed at a median of 2 weeks (IQR 2–2.5) post-end-of-treatment. Urine culture performed at 2 weeks post-end-of-treatment was available in 12 patients, with a microbiological cure in 11.

After a median of follow-up of 6 months (Q25–Q75, 4–12), 2 of 18 patients relapsed with an orchitis and with a new episode of ABP at 4 and 5 weeks post-end-of-treatment, respectively (Table 1).

## DISCUSSION

FT has been evaluated in urinary tract infections in different studies on non-prostatic infections (5, 8, 9). However, drawing conclusions about its utility in management of ABP is difficult because of the characteristics of the prostate.

There are some experiences reported with FT for treating CBP. Our group presented the results of 15 cases of difficult-to-treat CBP treated with FT (3  g/72 hours for 6 weeks); clinical cure was reported in seven (47%) of them (10). Karaiskos et al. also reported the outcomes of 44 patients with CBP treated with FT 3  g/48 hours for 6–12 weeks. Microbiological eradication was achieved in 34 patients (77%) (11). To our best knowledge, the only published evidence in ABP comes from the study of Bouiller et al. Three patients with ABP treated with oral FT 3 g/24 hours for 3 weeks were clinically and microbiologically cured, with no recurrence at 6 months (12). In our study, we used FT as step-down therapy after an initial period of intravenous beta-lactams. This lead-in phase is important because it could help to reduce the bacterial load and improve results. We achieved a clinical cure in all cases. We observed a recurrence in two cases, and in one of them, a lead-in phase of intravenous treatment was not done.

An unresolved point in the use of FT is the most suitable dose. Based on pharmacokinetic data (4, 12), some authors recommend daily FT treatment. It could be especially important in cases of microorganisms with high MIC, as occurs in *K. pneumoniae* strains (5). Nevertheless, this posology could be associated with a higher rate of adverse effects. On the other hand, a 3 g dose every 3 days seems to present a higher risk of failure (10). In our study, we use the dose of 3 g/48 hours with a good tolerance and acceptable clinical success. This may be so because of an improved penetration of antimicrobials in an inflamed prostate and because most of our patients received an initial treatment with intravenous beta-lactams.

This study has the limitations of an observational, single-center, non-comparative design with a small sample size, limiting the generalizability of the results. Moreover, diagnosis of ABP can be difficult, and there is a current lack of agreement on the diagnosis criteria. Finally, fosfomycin MIC determination using E-test was not available; as a consequence, we were unable to know the exact MIC values.

Despite these limitations, our study provides new data that help to consider FT as a plausible alternative for treating ABP in patients with resistance or side effects to first-line drugs. The availability of an alternative oral treatment to avoid the use of the carbapenems could have important benefits in terms of quality of life of the patient, health costs, and an ecological impact.
